# COVID-19 PCR: frequency of internal control inhibition in clinical practice

**DOI:** 10.1099/acmi.0.000478.v3

**Published:** 2023-05-19

**Authors:** Alessandro C. Pasqualotto, Amanda L. Seus

**Affiliations:** ^1^​ Federal University of Health Sciences of Porto Alegre (UFCSPA), Porto Alegre, Brazil; ^2^​ Molecular Biology Laboratory, Irmandade Santa Casa de Misericordia de Porto Alegre, Porto Alegre, Brazil; ^3^​ Universidade do Vale do Rio dos Sinos (UNISINOS), São Leopoldo, Brazil

**Keywords:** SARS-CoV-2, Covid-19, qPCR, inhibition, internal control

## Abstract

**Introduction.:**

Diagnosis of COVID-19 (coronavirus disease 2019) is best performed with real-time (quantitative) PCR (qPCR), the most sensitive method for detection and quantification of viral RNA. Using the Centers for Disease Control and Prevention (CDC) protocol, for each sample tested for the virus, three qPCR tests are performed, targeting the viral genes N1 and N2, in addition to the internal control gene RNase P. Samples in which internal control fails to amplify should be labelled ‘invalid’.

**Methods.:**

This study aims to determine the frequency of inhibition of the RNase P gene used as an internal control in qPCR tests for SARS-CoV-2 (severe acute respiratory syndrome coronavirus 2) in a reference hospital in Southern Brazil during the COVID-19 pandemic (1 February 2021 to 31 March 2021).

**Results.:**

A total 10, 311 samples were available for analysis. The mean cycle threshold (Ct) value for the RNAse P gene was 26.65 and the standard deviation was 3.18. A total of 252 samples were inhibited (2.4%) during the study period: amongst these, 77 (30.5%) showed late amplifications (beyond 2 standard deviations from the mean Ct value), and 175 (69.4%) revealed no fluorescence at all for the RNase P gene.

**Conclusions.:**

This study showed a low percentage of inhibition using RNase P as an internal control in COVID-19 PCRs using the CDC protocol, thus proving the effectiveness of this protocol for identification of SARS-CoV-2 in clinical samples. Re-extraction was efficacious for samples that showed little or no fluorescence for the RNase P gene.

## Data Summary

The authors made available all data obtained from all PCR runs performed during the months analyzed in the study in excel spreadsheet format.

## Introduction

In Brazil, coronavirus disease 2019 (COVID-19) disseminated quickly; only 18 months after the notification of its first reported occurrence in the country, more than 21 million cases have been reported, and more than 587000 patients have died of the disease [1]. Diagnosis of COVID-19 is best performed with real-time PCR (qPCR), the most sensitive method for detection and quantification of viral RNA [2]. The Centers for Disease Control and Prevention (CDC) established that for each sample tested for the virus, three qPCR tests should be performed, targeting the viral genes N1 and N2, in addition to the internal control gene RNase P [3]. For positivity, fluorescence curves should cross the cycle threshold (Ct) within the limit of 40 cycles. Failure to amplify the RNase P gene may indicate inadequate nucleic acid extraction, lack of sufficient human cells, inadequate setup and execution of the assay, or malfunction of the reagents/equipment. Samples in which internal control fails to amplify should be labelled ‘invalid’, and in such cases a new nucleic acid extraction should be performed [3]. Searching the literature, it is surprising how little information is available on the frequency of internal control inhibition in different centres. Thus, this study aims to determine the frequency of inhibition of the RNase P gene used as an internal control in qPCR tests for SARS-CoV-2 (severe acute respiratory syndrome coronavirus 2) in a reference hospital in Southern Brazil during the COVID-19 pandemic.

## Methods

All biological samples (mostly nasopharyngeal swab, sputum and liquor) tested for COVID-19 qPCR at the Molecular Biology Laboratory at Santa Casa de Misericordia de Porto Alegre, between 1 February 2021 and 31 March 2021, were considered for study. qPCR tests were performed according to the CDC protocol [3], in ABI 7500, StepOne or StepOne Plus thermocyclers. Samples were excluded if they were received outwith the predefined dates.

For nucleic acid extraction, three different methods were used: ReliaPrep Viral TNA Miniprep System (manual method using columns; Promega), Maxwell RSC Viral Total Nucleic Acid Purification Kit (semi-automated extraction based on magnetic beads; Promega) and Pure Link RNA Mini Kit (also a manual kit based on columns; Invitrogen).

To determine the frequency of internal control inhibition, we studied the Ct values of the RNAse P gene for each sample and compared that with the Ct value of the RNAse P gene for the whole population being tested. All samples amplifying with ≥2 standard deviations beyond the mean Ct value for the internal control gene were considered inhibited. We were also interested in studying the effect of re-amplification and/or re-extraction in the inhibited samples.

Descriptive statistics were used to summarize the data. The *P* values were considered statistically significant if ≤0.05. Data analysis was performed with SPSS 20.0 (IBM).

## Results

During the period of study, a total 11, 954 samples were processed in the laboratory, 4,164 were positive (34.8 %), 7,784 (65.1 %) were negative, and six were inconclusive or invalid (0.05 %). Among these, 10, 311 samples were available for analysis – 13 % were excluded due to loss of data.

The samples were considered invalid when there were pre-analytical problems (i.e., when the collection tubes did not contain the swab), and inconclusive when internal control failed to amplify in the initial qPCR run. These samples were submitted to re-extraction and/or re-amplification.

The mean Ct value for the RNAse P gene was 26.65 and the standard deviation was 3.18. A total of 252 samples were inhibited (2.4 %) during the study period: amongst these, 77 (30.5 %) showed late amplifications (beyond 2 standard deviations from the mean Ct value), and 175 (69.4 %) revealed no fluorescence at all for the RNase P gene.

As shown in Table 1, from the 175 samples that were totally inhibited for the RNAse P gene, 140 (80.0 %) were extracted with the ReliaPrep, 23 (13.1 %) with Maxwell and 12 (6.9 %) with Pure Link. Among those that showed late amplification for the RNAse P gene (*n*=77), 39 (50.6 %) were extracted with ReliaPrep and 38 (49.3 %) with Maxwell.

**Table 1.** Corrective actions taken and test result according to nucleic acid extraction method and type of inhibition

	Total samples	Re-extracted	Re-amplified	No correction action (NCA)	Re-extracted and re-amplified	Positive	Negative	Inconclusive	Invalid
**Totally inhibited**									
ReliaPrep	140	26	64	16	34	35	103	1	1
Maxwell	23	12	5	6	0	8	14	1	0
Pure Link	12	0	0	0	12	0	12	0	0
**Late amplification**									
ReliaPrep	39	10	10	19	0	6	32	0	1
Maxwell	38	9	2	27	0	9	29	0	0
Pure Link	0	0	0	0	0	0	0	0	0
**Total**	**252**	**57**	**81**	**68**	**46**	**58**	**190**	**2**	**2**

Of the 252 inhibited samples, 68 were reported without any corrective action, 20 because these were positive samples for the novel coronavirus, 34 were negative but they showed late amplification, two were invalid and the other 12 were negative and did not undergo any measures of correction. On the other hand, 103 were submitted to nucleic acid re-extraction, resulting in 30 positive results for COVID-19 (29.1 %), 71 negative results (68.9 %) and two inconclusive results (1.9 %). Also, 81 samples were re-amplified (and not re-extracted), and eight of these were found to be positive for COVID-19 (9.9 %).

## Discussion

The RNase P gene is used in the CDC protocol as an internal control for detection of COVID-19, since this is a multi-copy gene that is abundant in the human genome, and therefore is easily detectable [4]. Despite the widespread use of qPCR tests in clinical medicine, it was surprising that at the time of writing of this manuscript there was a limited amount of data produced in the literature on the frequency of PCR inhibitors in clinical practice. Articles were found comparing pathogen-specific controls with human gene amplification [5–7], but none of them related to COVID-19.

Döskaya *et al*. [8] determined the degree and frequency of inhibition in qPCR for the detection of *Pneumocystis jirovecii* in respiratory samples. That differed from our work, which used all types of incoming biological samples and worked with SARS-CoV-2. In addition, the inhibition frequency in their study was 26.3 %, much higher than that in our study (2.4 %). Furthermore, the corrective measure for qPCR inhibition was dilution of the samples, whilst in our study samples were re-extracted or re-amplified. As can be seen from our results, the measures used in the laboratory were efficient, given that only two results were inconclusive and two invalid, totalling 1.58 % (4/252) of all samples that were found to be inhibited in the PCR test. The two invalid samples did not have swabs inside the tube used for sample collection, and therefore there was probably no DNA for analysis.

Moreover, the focus on minimizing the impact of false-negative results is increasing (i.e. assuming negative results truly represent the absence of qPCR diagnostic targets [9]), and therefore 103 samples were subjected to re-extraction and 128 to re-amplification. In addition, when the Maxwell method was used, the percentage of partially inhibited samples was higher than with the fully inhibited methods, showing that it has high recovery of the sample DNA.

## Conclusions

This study showed a low percentage of inhibition using RNase P as an internal control in COVID-19 PCRs using the CDC protocol, particularly when the Maxwell system is used for nucleic acid extraction. Inhibitions can be promptly resolved by submitting samples to re-extraction, which suggests that inadequate mixing of sample with lysate buffer may account for most of the inhibited samples. We have therefore shown the effectiveness of this protocol for the identification of SARS-CoV-2 through the qPCR technique and of re-extracting and amplifying samples that showed little or no fluorescence for the RNase P gene.

## Supplementary Data

Supplementary material 1
